# Predictive accuracy of population meropenem models in critically ill pneumonia patients

**DOI:** 10.1128/aac.00458-26

**Published:** 2026-07-10

**Authors:** Adrian Valadez, Emma Harlan, Colin Jeantet, Marc H. Scheetz, Michael N. Neely, Helen K. Donnelly, Richard G. Wunderink, Nathaniel J. Rhodes

**Affiliations:** 1 Department of Pharmacy Practice, Midwestern University, College of Pharmacy 15475, Downers Grove, Illinois, USA; 2 Pharmacometrics Center of Excellence, Midwestern University 69281, Downers Grove, Illinois, USA; 3 Department of Pharmacy, Northwestern Memorial Hospital 24560, Chicago, Illinois, USA; 4 Department of Biomedical Sciences, College of Graduate Studies, Midwestern University, Downers Grove, Illinois, USA; 5 Department of Pharmacology, College of Graduate Studies, Midwestern University, Downers Grove, Illinois, USA; 6 Keck School of Medicine, University of Southern California 12223 , Los Angeles, California, USA; 7 Laboratory of Applied Pharmacokinetics and Bioinformatics, The Saban Research Institute, Children's Hospital of Los Angeles 466592, Los Angeles, California, USA; 8 Division of Pulmonary and Critical Care Medicine, Department of Medicine, Northwestern University Feinberg School of Medicine 12244, Chicago, Illinois, USA; University of Houston, Houston, Texas, USA

**Keywords:** pharmacokinetics, pneumonia, external validation, bayesian model, renal replacement

## Abstract

Population pharmacokinetic (PK) models may not generalize across critically ill populations. We evaluated the transportability of published parametric and nonparametric meropenem models as Bayesian priors. Seven published meropenem models were implemented in Monolix 2024R1. Nonparametric models were converted to log-normal priors from reported medians and CV%. An independent cohort was used for evaluation. *A priori* and *a posteriori* predictions were summarized across eligible observations and separately for observations obtained during continuous renal replacement therapy (CRRT) and in the absence of CRRT. Predictive performance was assessed using relative mean prediction error (rMPE) and relative median absolute prediction error (rMdAPE). An rMPE within ±20% and an rMdAPE ≤30% were defined as acceptable. Seventeen patients, including six receiving CRRT, contributed 74 plasma concentrations. In the pooled cohort, only the Rohani and Shekar models met predefined performance thresholds at both population and individual levels (*a priori* rMdAPE 24.6%–26.4%; *a posteriori* rMdAPE 6.7%–11.1%). In CRRT patients, no model achieved acceptable *a priori* performance; after Bayesian updating, all CRRT-specified models met accuracy and precision criteria, with the nonparametric models (Reed and Rohani) demonstrating low imprecision. In non-CRRT patients, several models showed population-level bias, but all met predefined accuracy and precision thresholds after Bayesian updating. Among evaluated models, Rohani, Reed, and Shekar demonstrated the most consistent predictive performance across the CRRT and non-CRRT subgroups. Nonparametric model summaries can be translated into parametric priors, but model selection remains critical when applying Bayesian dosing in critically ill patients.

## INTRODUCTION

Meropenem is a broad-spectrum β-lactam antibiotic commonly used for the treatment of hospital-acquired and ventilator-associated pneumonia in critically ill patients. Profound pathophysiological alterations in critical illness (e.g., expanded volume of distribution, altered renal clearance, and frequent use of extracorporeal organ support) can lead to substantial pharmacokinetic (PK) variability and unpredictable drug exposure when standard dosing strategies are applied (1). Fixed dosing regimens may therefore lead to subtherapeutic or excessive concentrations, particularly in patients with augmented renal clearance or renal replacement therapy exposure (2). These challenges have motivated increasing use of therapeutic drug monitoring (TDM) and individualized dosing strategies to optimize β-lactam exposure in the intensive care unit (ICU) (3). Population PK models provide a quantitative framework to characterize drug disposition and inter-individual variability and are increasingly incorporated into model-informed precision dosing (MIPD) platforms. However, a persistent challenge in clinical pharmacometrics is model transportability, the extent to which a model developed in one population maintains predictive performance when applied to a different external cohort (4). Critically ill patients represent a particularly complex population for model transport, given heterogeneity in renal function, dynamic physiology, and variable exposure to renal replacement therapy or extracorporeal support. Prior external evaluations of antibiotic PK models have demonstrated that predictive performance may degrade when models are applied outside their derivation setting, underscoring the need for rigorous external validation prior to clinical implementation (4–6).

Several meropenem population PK models have been developed in critically ill and renally impaired populations, incorporating differing structural assumptions and covariate relationships for renal function and organ support (7–13). Whether these models retain adequate predictive performance when transported into a pneumonia-focused ICU cohort that includes patients receiving continuous renal replacement therapy (CRRT) remains uncertain.

The objective of this study was to evaluate the predictive performance and transportability of published parametric and translated nonparametric meropenem population PK models when applied as fixed Bayesian priors, without parameter re-estimation, to an external cohort of critically ill pneumonia patients with and without CRRT. To contextualize performance, comparator meropenem models derived in distinct but clinically relevant populations were evaluated in parallel. This study therefore examines the reliability of published meropenem PK models for supporting Bayesian precision dosing in critically ill pneumonia patients.

## MATERIALS AND METHODS

### Study design and patient population

This external evaluation utilized plasma samples collected through the Successful Clinical Response in Pneumonia Therapy (SCRIPT) study, a prospective observational study conducted at a single academic medical center (14). Adult ICU patients treated with meropenem for pneumonia who contributed at least one quantifiable plasma meropenem concentration were eligible for inclusion. Patients were sequentially enrolled through the parent prospective observational SCRIPT study, and the present evaluation included the subset with available quantifiable meropenem concentrations, complete dosing histories, and clinical covariates required for external model implementation. Thus, cohort selection was based on the availability of evaluable samples and required clinical data, rather than random sampling or targeted selection of a specific clinical phenotype. These data were not included in the development of the previously published population PK models evaluated in this analysis. Required data included dose amount, infusion start and stop times, sample collection times, body size descriptors, serum creatinine, estimated renal function, and renal replacement therapy status and settings when applicable.

Clinical covariates required for model implementation included age, sex, body weight, serum creatinine, estimated creatinine clearance (Cockcroft-Gault) (15), and renal replacement therapy (RRT) modality and settings. Clinical data, complete dosing histories, and RRT records were extracted from the electronic medical record. For models incorporating CRRT-specific clearance, CRRT exposure was encoded using time-varying RRT records, including CRRT start and stop times and available CRRT settings during the dosing and sampling intervals.

Observations were classified according to renal support status at the time of sampling. Samples obtained while a patient was receiving CRRT were classified as CRRT, irrespective of prior intermittent hemodialysis (HD) exposure. Patients receiving intermittent HD without concurrent CRRT at the time of sampling were excluded by design. Observation-level CRRT classification was used only for a stratified summary of predictive performance.

Meropenem was administered according to routine clinical practice, typically as 1 g or 2 g every 8 h delivered as prolonged infusions (approximately 3 h) with loading doses of 1 g or 2 g administered at clinician discretion. Blood samples were collected during routine clinical care. Plasma samples were held at 2°C–4°C for no more than 48 h prior to storage at −80^°^C. Sample times were recorded relative to infusion start and stop times.

### Bioanalytical assay

Plasma meropenem concentrations were quantified using a validated liquid chromatography-tandem mass spectrometry (LC-MS/MS) assay performed on an Agilent 6420A triple quadrupole mass spectrometer. The assay had a lower limit of quantification (LLOQ) of 1 mg/L and was linear over a range of 1–100 mg/L (R^2^ ≥0.997).

Intra-day accuracy ranged from 91% to 110%, with corresponding precision (coefficient of variation, CV%) of 0.18%–1.95%. Inter-day accuracy ranged from 86% to 104%, with precision ≤6%. These performance characteristics were consistent with accepted bioanalytical method validation criteria. (16).

Meropenem (m/z 384.1) was monitored using product and qualifier ions of 68.2 and 141, respectively. Ceftazidime (m/z 274.1) was used as the internal standard and monitored using product and qualifier ions of 80.2 and 126, respectively.

Chromatographic separation was performed using an Agilent InfinityLab Poroshell 120 ECC-18 column (3.0 × 100 mm, 2.7 µm) maintained at 25°C. The mobile phase consisted of Milli-Q water (solvent A) and LC-MS grade acetonitrile (solvent B) (17). A step-gradient was used at a flow rate of 0.7 mL/min: 90% A/10% B for 1.5 min; increase to 95% B through 3.5 min; returned to initial conditions at 3.51 min; and re-equilibrated through 6.0 min. Retention times were approximately 1.93 min for meropenem and 1.63 min for ceftazidime.

### Population pharmacokinetic model implementation

All population PK models were implemented in Monolix (version 2024R1) (18) using the structural equations, covariate relationships, and published population parameter estimates reported in the original manuscripts. Fixed-effect parameters (ϑ) and between-subject variability terms (ω) were fixed to published values. No parameter re-estimation or covariate re-selection was performed.

For each model, two prediction models were evaluated: (i) population (*a priori*) predictions based on fixed priors and individual covariates, and (ii) individual (*a posteriori*) predictions obtained using maximum *a posteriori* (MAP) estimation (empirical Bayes estimates).

Continuous and categorical covariates were implemented according to the functional forms specified in the source publications.

### Variability translation and covariate reconstruction

When between-subject variability was reported as coefficient of variation (CV%), values were converted to log-normal standard deviations (ω) for implementation in Monolix using established relationships. (19).

Renal function covariates not directly available in the external data set were reconstructed using standard estimating equations consistent with the source publications. Estimated glomerular filtration rate (eGFR) was calculated using the four-variable Modification of Diet in Renal Disease (MDRD) equation as originally described by Levey et al. (20). When required, eGFR was also calculated using the Chronic Kidney Disease Epidemiology Collaboration (CKD-EPI) equation as described by Levey et al. (21).

Both equations were implemented using available serum creatinine, age, and sex variables. Race-based coefficients were omitted because race was unavailable in the external data set. Residual diuresis, when required, for the O’Jeanson model, was extracted from the electronic medical record. Because residual diuresis may not always be available or consistently documented in routine clinical workflows, two implementations of the O’Jeanson model were evaluated: (i) O’Jeanson (individual RD), incorporating residual diuresis values extracted from the electronic medical record; and (ii) O’Jeanson (fixed RD), in which residual diuresis was fixed to the development population median.

### Primary model: Rohani et al.

The primary model evaluated was the meropenem population PK model developed by Rohani et al. (12). This model was implemented using published structural and covariate specifications, including nonlinear scaling of intrinsic renal clearance with creatinine clearance and explicit clearance components for CRRT. All fixed-effect parameters and variability terms were applied without modification. Covariate equations and parameter values are provided in the Supplementary Materials.

### Comparator population PK models

Comparator meropenem PK models were implemented as reported in their original publications, using fixed-effect and variability parameters without re-estimation (7–11, 13). Candidate models were selected *a priori* using a literature-based, implementation-focused approach rather than a systematic review of all published meropenem population PK models. Published models were considered when they had previously been evaluated in critically ill adults, including CRRT-focused external evaluations, or when they were clinically relevant to the present ICU pneumonia cohort. Additional literature searches were performed to identify nonparametric meropenem population PK models. Nonparametric models were included when the source publication provided sufficient structural, covariate, fixed-effect, and variability information to support translation into parametric prior distributions for implementation within the common Bayesian forecasting framework.

The pooled analysis included all eligible observations combined but was restricted to models structurally capable of representing both CRRT and non-CRRT conditions. Stratified analyses were then performed separately for observations obtained during CRRT and observations obtained in the absence of CRRT. Models that did not include a CRRT- or RRT-specific clearance mechanism were restricted to the non-CRRT subgroup.

All evaluated models, including structural characteristics, fixed-effect parameters (ϑ), between-subject variability terms (ω), and residual unexplained variability terms (RUV), are summarized in Tables S1 and S3. Renal clearance equations used for model translation are summarized in Table S4, and detailed covariate equations are provided in the Supplementl material. A supplementary cohort-comparison table was added to summarize available development-population descriptors for each evaluated model, including renal function, renal replacement therapy exposure, body size, illness severity, and other reported model-relevant covariates; descriptors not reported in the source publication were listed as not reported (Table S5).

Models originally developed using nonparametric estimation were translated to parametric form using published population summaries to enable implementation within a unified Bayesian forecasting framework.

### Predictive performance metrics

Predictive performance was evaluated using prediction-based diagnostics (22). Analyses were conducted in the pooled cohort and stratified by renal support status (CRRT vs. non-CRRT).

For each observed plasma meropenem concentration, two sets of predictions were generated for each model:

(i) Population (*a priori*) predictions based on population priors and individual covariates.
(ii) Individual (*a posteriori*) predictions obtained using maximum *a posteriori* (MAP) estimation after incorporation of observed concentrations.

Relative prediction error (rPE) for each observation was defined as:


$$rPE_{ij}=\;\frac{C_{ij}^{pred}-\;C_{ij}^{obs}}{C_{ij}^{obs}}\;x\;100$$

where C*_ij_*^obs^ represents the observed concentration for the subject *i* at time *j*, and C*_ij_*^pred^ represents the corresponding predicted concentration.

Prediction bias was quantified using the relative mean prediction error (rMPE):


$$rMPE=\;\frac{1}{n}\sum_{i,\;j}rPE_{ij}$$

Prediction imprecision was quantified using the relative median absolute prediction error (rMdAPE):


$$rMdAPE=median\;\left(|rPE_{ij}|\right)$$

Models were considered acceptable when the relative mean prediction error (rMPE) was within ±20%, and the relative median absolute prediction error (rMdAPE) was ≤30%. These prespecified criteria were applied to both population (*a priori*) and individual (*a posteriori*) predictions in pooled, CRRT, and non-CRRT analyses.

### Model diagnostics and statistical analysis

Patients receiving intermittent hemodialysis without concurrent CRRT at the time of sampling were excluded by design, as analyses were stratified by CRRT exposure (CRRT vs. non-CRRT). Patients sampled while receiving CRRT were classified as CRRT irrespective of prior intermittent HD exposure.

Model diagnostics included observed-versus-predicted (OP) concentration plots at both the population (*a priori*) *and* individual (*a posteriori*) levels, as well as individual weighted residuals (IWRES) plotted against time and individual predictions. OP plots were generated separately for the pooled cohort and stratified by CRRT exposure to evaluate model bias and dispersion within the CRRT and non-CRRT subgroups. All predictive performance metrics and graphical diagnostics were generated using R (version 4.5.1) (23).

## RESULTS

### Patient characteristics and data set

Seventeen ICU patients treated with meropenem for pneumonia contributed 74 plasma meropenem concentrations for evaluation. One additional concentration from a CRRT-treated patient was excluded before predictive performance analyses because it was discordant with the documented dosing history and surrounding concentration-time profile; specifically, the sample was obtained approximately 2 h after a recorded dose, yet was inconsistent with other concentrations from the same patient at comparable post-dose times. No documentation error could be identified. Six patients were receiving CRRT at the time of sampling, contributing 31 observations; the remaining 43 observations were obtained from non-CRRT patients. Baseline demographics and clinical characteristics are summarized in Table 1.

**TABLE 1:** Baseline demographics and clinical characteristics (*N* = 17)^*a*^

Demographics	Mean ± SD or N (%)
Age (years)	64.4 ± 15.9
Weight (kg)	80.6 ± 22.2
BSA (m^2^)	1.9 ± 0.3
SCr (mg/dL)	1.7 ± 1.2
CrCl (mL/min)	76.8 ± 66.2
CKD-EPI (mL/min/1.73 m^2^*)*	59.2 ± 34.5
MDRD (mL/min/1.73 m^2^*)*	67.5 ± 13.2
Urine output (DIUR) in mL/day	1240.1 ± 1593.1
SOFA score	11.7 ± 4.2
CRRT, n (%)	6 (35%)
Sex, n (%)	
Male	12 (71%)
Female	5 (29%)

^*a*^ TBW, total body weight; BSA, body surface area; CrCl, creatinine clearance; CRRT, continuous renal replacement therapy; DIUR, residual diuresis; SCr, serum creatinine; SOFA, Sequential Organ Failure Assessment. CKD-EPI and MDRD estimates were calculated without race adjustment due to unavailable race data.

### Pooled predictive performance across published models

**Fig 1:**
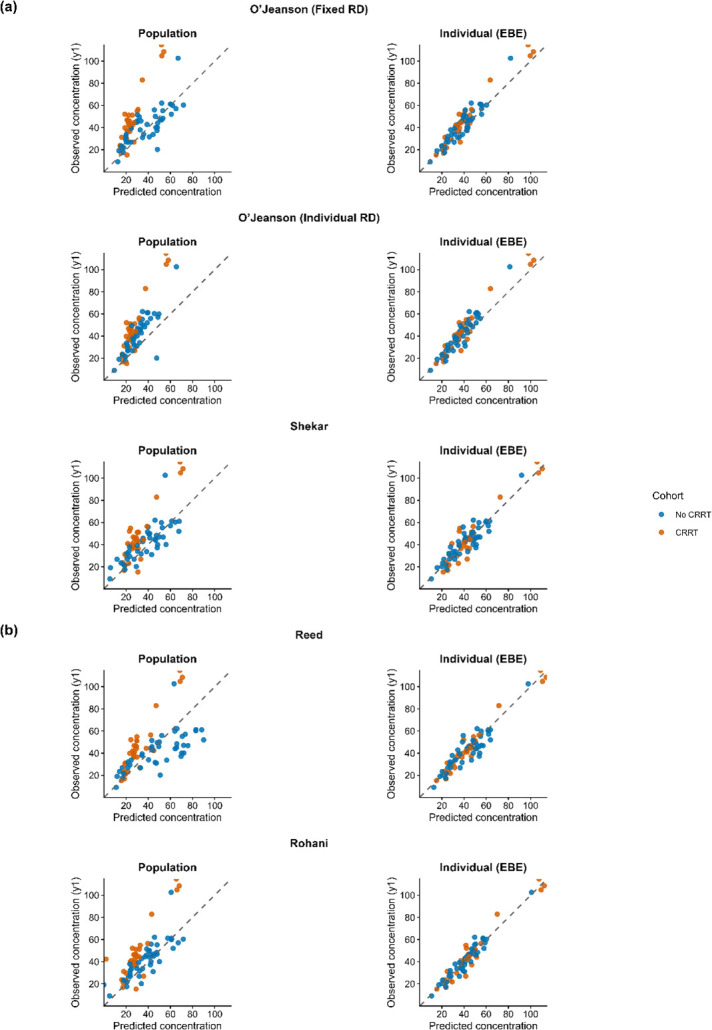
Observed versus predicted meropenem concentrations across published population PK models. Population predictions (left panel of each model) and individual Bayesian predictions (right panel) are shown for both parametric panel (**a**) and nonparametric panel (**b**) model subsets. The dashed line represents the line of identity. Points are colored by renal replacement therapy (RRT) status.

In the pooled cohort (*n* = 74), predictive performance differed across the five model implementations evaluated: O’Jeanson (individual RD), O’Jeanson (fixed RD), Reed, Rohani, and Shekar. (Table 2 and Fig. 1).

**TABLE 2:** Pooled predictive performance across published models^*a*^

All observations (*n* = 74)					
	Models	Population		Individual	
		rMPE (%)	rMdAPE (%)	rMPE (%)	rMdAPE (%)
Parametric models	O’Jeanson (fixed RD)	−19.0	33.2	−7.5	13.7
	O’Jeanson (individual RD)	−27.2	32.2	−8.1	14.0
	Shekar	−15.7	26.4	1.7	11.1
Nonparametric models	Reed	−0.3	32.5	3.4	7.7
	Rohani	−16.5	24.6	2.5	6.7

^*a*^ Predictive performance of published meropenem population PK models evaluated against observed concentrations in the pooled cohort. Relative mean prediction error (rMPE) and relative median absolute prediction error (rMdAPE) are reported for population and individual (Bayesian) predictions. Positive rMPE values indicate overall overprediction, whereas negative values indicate underprediction.

At the population (*a priori*) level, only Rohani and Shekar met the prespecified acceptability thresholds (Rohani: rMPE −16.5%; rMdAPE 24.6%; Shekar: rMPE −15.7%; rMdAPE 26.4%). Reed demonstrated low bias but moderate precision (rMPE −0.3%; rMdAPE 32.5%). Both O’Jeanson implementations showed substantial underprediction, with greater population-level bias observed when individual residual diuresis was incorporated (O’Jeanson [individual RD]: rMPE −27.2%; O’Jeanson [fixed RD]: rMPE −19.0%).

Following Bayesian updating, individual (*a posteriori*) predictions showed improved accuracy and precision across all models. All models met the predefined performance thresholds at the individual level. Shekar exhibited the lowest residual bias (rMPE 1.7%), whereas the translated nonparametric-derived priors Rohani and Reed demonstrated acceptable precision (rMdAPE 6.7% and 7.7%, respectively) and low bias (Rohani: rMPE 2.5%; Reed: rMPE 3.4%). Both implementations of the O’Jeanson model also demonstrated acceptable Bayesian posterior bias and precision (O’Jeanson [fixed RD]: rMPE −7.5%; rMdAPE 13.7%; O’Jeanson [individual RD]: rMPE −8.1%; rMdAPE 14.0%). Observed-versus-predicted plots showed tight clustering around the line of identity for Rohani, Reed, and Shekar in the pooled analysis (Fig. 1).

### Predictive performance in the CRRT subgroup

Among patients receiving CRRT (31 observations), population-level performance was reduced for all five CRRT-specified models (O’Jeanson [individual RD], O’Jeanson [fixed RD], Reed, Rohani, and Shekar) (Table S2 and Fig. S1). None of the models met the predefined performance thresholds at the population level. All implementations demonstrated underprediction with moderate to high imprecision (O’Jeanson [individual RD]: rMPE −35.2%; rMdAPE 41.1%; O’Jeanson [fixed RD]: rMPE −39.7%; rMdAPE 45.1%; Shekar: rMPE −25.7%; rMdAPE 34.0%; Reed: rMPE −26.6%; rMdAPE 33.7%; and Rohani: rMPE −28.6%; rMdAPE 37.1%).

Bayesian updating substantially improved individual-level predictions across models in the CRRT subgroup. All five models met the predefined performance thresholds after updating, with low residual bias and rMdAPE values ≤ 15.0% (O’Jeanson [individual RD]: rMPE −8.2%; rMdAPE 13.5%; O’Jeanson [fixed RD]: rMPE −8.9%; rMdAPE 14.2%; Shekar: rMPE 3.4%; rMdAPE 11.8%; Reed: rMPE 1.7%; rMdAPE 5.0%; and Rohani: rMPE 3.6%; rMdAPE 5.1%). The lowest imprecision was observed for the Reed and Rohani models. Observed-versus-predicted plots in CRRT patients demonstrated tighter clustering around the line of identity following Bayesian updating (Fig. S1).

### Predictive performance in the non-CRRT subgroup

In the non-CRRT subgroup (43 observations), predictive performance was evaluated using eight models (Table S2 and Fig. S2). At the population level, four models met the predefined performance thresholds: O’Jeanson (fixed RD), Reed, Rohani, and Shekar. O’Jeanson (fixed RD) showed low bias and acceptable precision (rMPE −4.1%; rMdAPE 18.6%), while Shekar, Reed, and Rohani also satisfied the prespecified accuracy and precision criteria (Shekar: rMPE −8.5%; rMdAPE 20.4%; Reed: rMPE 18.7%; rMdAPE 28.6%; and Rohani: rMPE −7.7%; rMdAPE 18.0%). In contrast, O’Jeanson (individual RD) exceeded the bias threshold despite acceptable precision (rMPE −21.5%; rMdAPE 28.3%). The Gijsen, Grijalba, and Li models showed substantial imprecision and/or underprediction at the population level (Gijsen: rMPE −3.5%; rMdAPE 42.0%; Grijalba: rMPE −24.8%; rMdAPE 31.1%; Li: rMPE −53.6%; rMdAPE 51.1%).

After Bayesian updating, individual-level predictions improved across all models in the non-CRRT subgroup, and all eight models met the predefined performance thresholds. Translated nonparametric-derived priors demonstrated the lowest residual imprecision, with Grijalba, Reed, and Rohani achieving rMdAPE values < 10.0% (Grijalba: rMPE 2.0%; rMdAPE 7.4%; Reed: rMPE 4.5%; rMdAPE 9.0%; and Rohani: rMPE 1.7%; rMdAPE 7.9%). Parametric models also achieved low residual bias and acceptable precision following updating (Gijsen: rMPE 1.3%; rMdAPE 19.2%; Li: rMPE −3.4%; rMdAPE 12.8%; O’Jeanson [fixed RD]: rMPE −6.6%; rMdAPE 13.6%). Observed-versus-predicted plots in non-CRRT patients reflected these findings, with closest alignment to the line of identity for individual predictions across models (Fig. S2).

### Individual parameter variability

**Fig 2:**
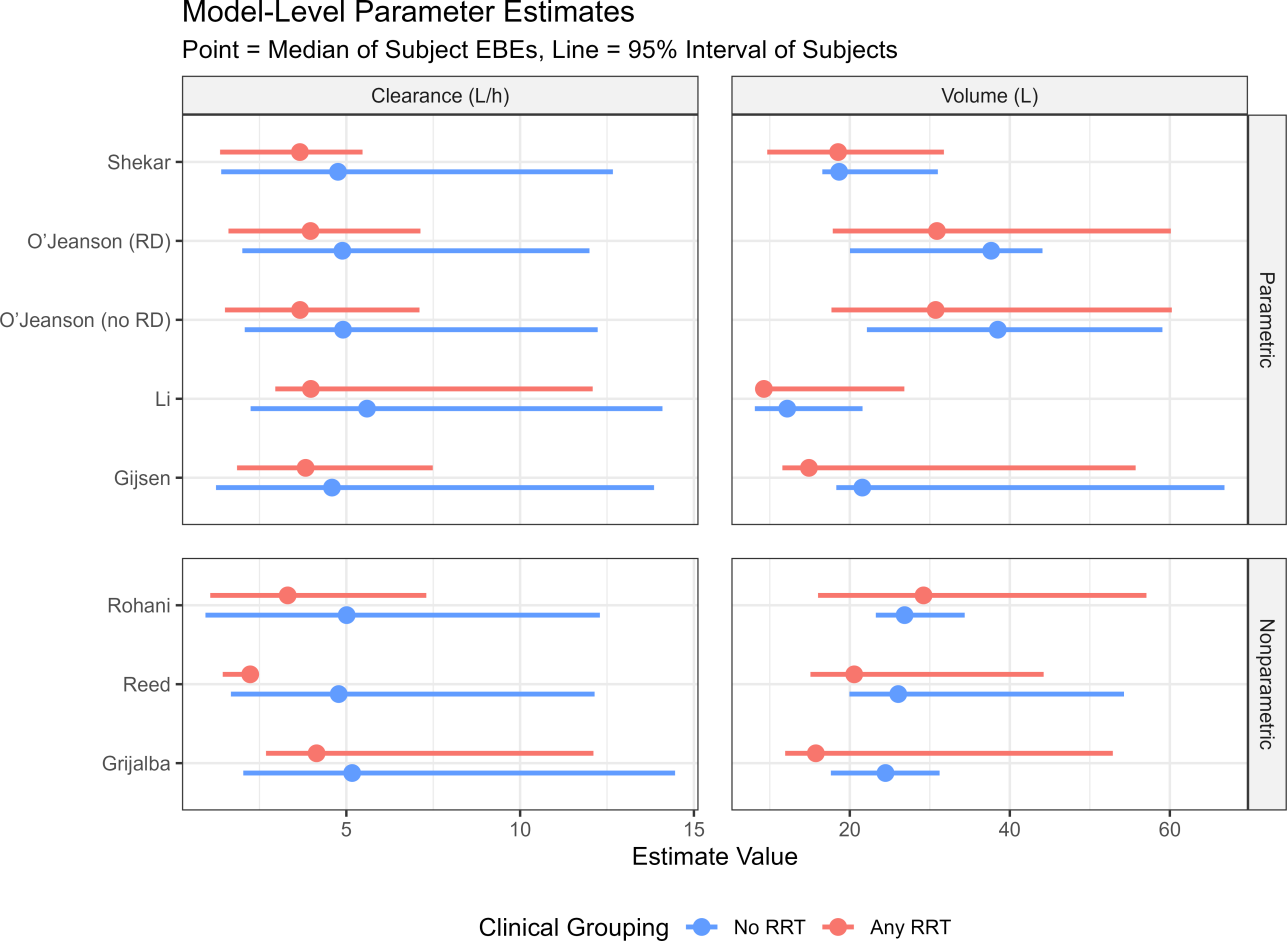
Distribution of individual clearance and volume estimates by model and renal support status. Median empirical Bayes estimates (EBEs) of clearance (CL, L/h) and volume (V, L) are shown for each evaluated population PK model. Points represent the median of subject-specific parameter estimates, and horizontal lines denote the 95% interval of subjects. Estimates are stratified by renal replacement therapy (RRT) status at the time of sampling (No RRT vs. Any RRT) and grouped by estimation paradigm (parametric and nonparametric).

**Fig 3:**
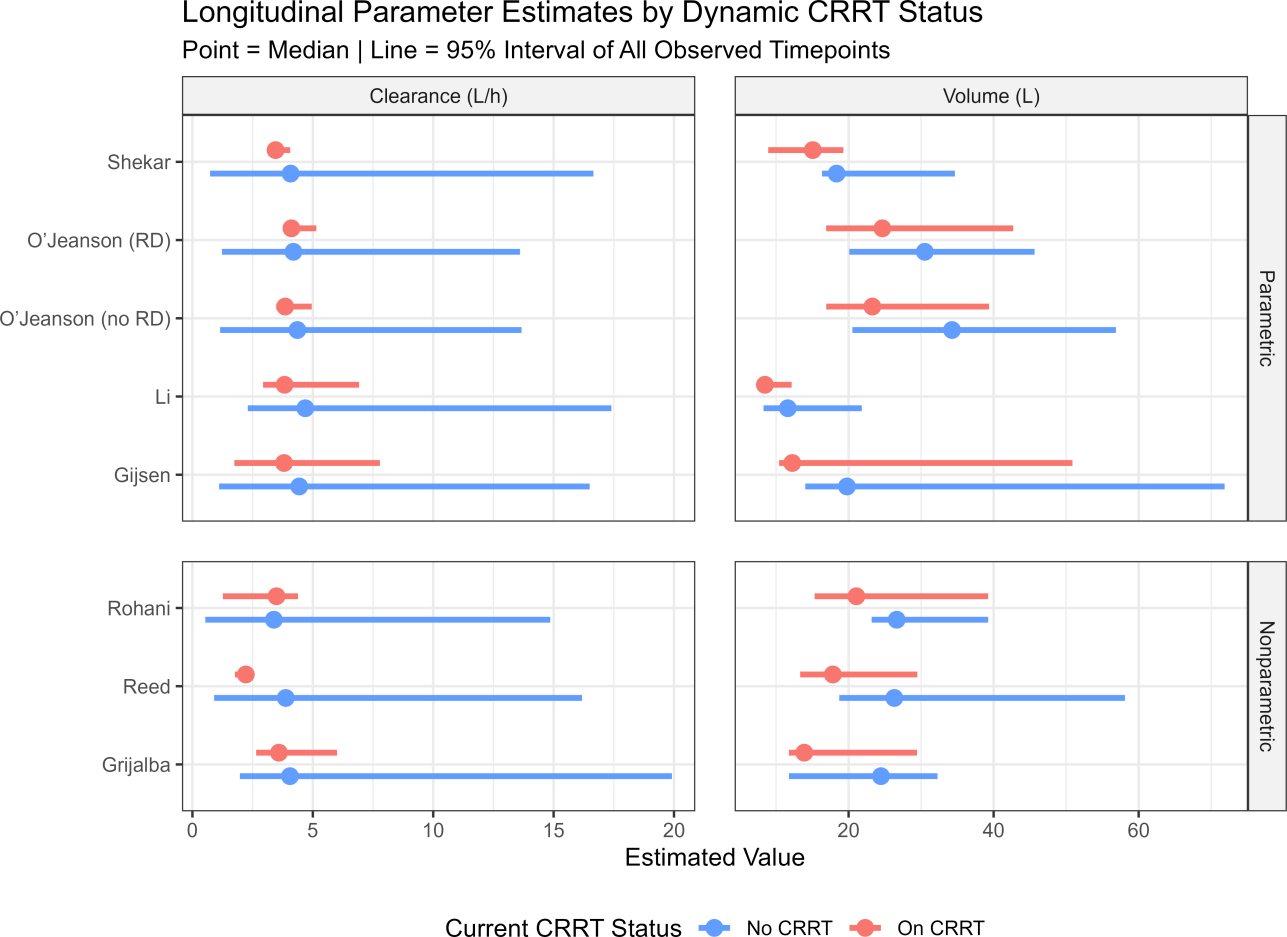
Longitudinal clearance and volume estimates stratified by CRRT status. Median empirical Bayes estimates of clearance (CL, L/h) and volume (V, L) are shown across all observed time points for each evaluated population PK model. Points represent the median parameter estimate, and horizontal lines denote the 95% interval across all time points. Estimates are stratified by concurrent CRRT status at the time of sampling (No CRRT vs. On CRRT) and grouped by estimation paradigm (parametric vs. nonparametric).

Individual parameter variability was first quantified numerically using subject-level empirical Bayes estimates (EBEs) summarized in Table 3. These quantitative findings are reflected visually in Fig. 2 and 3, which display the corresponding distributions of CL and V EBEs at the model level and across dynamic CRRT status. IWRES plots did not demonstrate systematic trends over time or across the range of individual predicted concentrations, suggesting no clear residual pattern favoring parametric or translated nonparametric-derived priors (Fig. S3 and S4).

**TABLE 3:** Individual-patient variability (CV%) in estimated meropenem PK parameters^*a*^^,^^*b*^

All patients (*n* = 17)					
	Models	Clearance		Volume	
		Mean	CV%	Mean	CV%
Parametric models	Gijsen	5.3	68.1	28.1	63.0
	Li	5.9	63.2	13.2	44.6
	O’Jeanson (fixed RD)	5.2	57.0	37.2	35.7
	O’Jeanson (individual RD)	5.1	57.0	34.2	32.2
	Shekar	4.9	66.2	19.6	30.8
Nonparametric models	Grijalba	5.9	70.0	23.9	43.1
	Reed	4.2	75.7	28.7	39.2
	Rohani	4.8	70.9	29.5	32.4

^*a*^ Individual Bayesian post hoc estimates of meropenem clearance and volume across the external evaluation cohort. Values are summarized as mean parameter estimates and corresponding coefficient of variation (CV%) for each evaluated parametric and nonparametric population PK model.

^*b*^ RD, residual diuresis; PK, pharmacokinetic.

For clearance (CL), translated nonparametric-derived priors demonstrated consistently greater between-subject dispersion than native parametric priors. Clearance coefficients of variation (CV%) ranged from 70.0% to 75.7% among translated nonparametric-derived priors (Grijalba 70.0%; Rohani 70.9%; and Reed 75.7%) compared with 57.0%–68.1% among native parametric priors (O’Jeanson [fixed RD] 57.0%; O’Jeanson [individual RD] 57.0%; Li 63.2%; Shekar 66.2%; Gijsen 68.1%) (Table 3). There was no overlap between these ranges, indicating systematically greater dispersion in clearance EBEs among translated nonparametric-derived priors.

In contrast, variability in volume (V) EBEs did not separate cleanly by estimation paradigm. Volume (CV%) ranged from 32.4% to 43.1% among translated nonparametric-derived priors (Rohani 32.4%; Reed 39.2%; and Grijalba 43.1%), whereas native parametric priors spanned a broader range of 30.8%–63.0% (Shekar 30.8%; O’Jeanson [individual RD] 32.2%; O’Jeanson [fixed RD] 35.7%; Li 44.6%; and Gijsen 63.0%). Thus, while clearance variability was consistently higher in translated nonparametric-derived priors, volume variability was more heterogeneous and overlapped substantially between paradigms.

## DISCUSSION

In this external evaluation of published meropenem population pharmacokinetic models applied to an independent cohort of critically ill pneumonia patients, the principal finding was that population-only predictions were insufficient in the most clinically complex subgroup: no CRRT-specified model achieved acceptable *a priori* performance in patients receiving CRRT, whereas Bayesian updating improved all CRRT-specified models to acceptable individual-level performance. Our analysis is unique in that published meropenem models were evaluated as fixed Bayesian priors in a pneumonia-focused ICU cohort with and without CRRT, without parameter re-estimation. This addresses a practical MIPD question: whether Bayesian updating can produce reliable individual predictions when published priors are transported into a clinically relevant target population. Observed concentrations improved prediction accuracy and precision across models; however, posterior performance remained model dependent, indicating that TDM can partially compensate for prior model misspecification but does not make all published priors interchangeable. Another unique aspect was the translation of nonparametric model summaries into parametric prior distributions, allowing nonparametric-derived and native parametric models to be evaluated within a common Bayesian forecasting framework. The Rohani, Reed, and Shekar models demonstrated the most consistent performance, supporting the importance of selecting candidate priors based on alignment with the intended population, particularly with respect to pneumonia ICU patients, renal replacement therapy representation, and renal-function covariates (24).

A defining feature of this study is the simultaneous evaluation of models developed under different statistical paradigms. Models originally developed using nonparametric estimation (Rohani, Reed, and Grijalba) were translated into parametric priors from published population summaries and evaluated alongside native parametric models (O’Jeanson, Shekar, Gijsen, and Li) without parameter re-estimation. This cross-paradigm implementation without re-estimation allowed performance differences to reflect structural and population alignment rather than re-fitting (25). Consistent with this framework, empirical Bayes-based variability summaries demonstrated higher clearance CV% for translated nonparametric-derived priors than for native parametric priors, whereas volume CV% overlapped more broadly between paradigms.

From a Bayesian perspective, each population model functioned as an informative prior, and incorporation of observed concentrations consistently reduced bias and imprecision relative to population predictions across models and strata. However, posterior updating did not eliminate differences between models, underscoring that prior structure still influenced posterior performance. Residual diagnostics, including IWRES-versus-time and IWRES-versus-prediction plots, did not reveal major temporal trends or heteroscedasticity, suggesting that systematic discrepancies between models were driven primarily by structural and covariate differences rather than residual error misspecification.

Performance differences were most evident when models were applied outside the clinical contexts in which they were developed. Models lacking explicit CRRT-specified clearance mechanisms performed poorly at the population level in CRRT patients and were appropriately restricted to non-CRRT analyses. Conversely, models incorporating renal replacement therapy structure (Rohani, Reed, and Shekar) were suitable for CRRT-stratified evaluation and demonstrated acceptable individual-level performance after updating. Within a given structural framework, covariate reconstruction also influenced performance: the two O’Jeanson implementations, which differed only in the handling of residual diuresis (individual values versus fixed population median), produced materially different population-level bias in both CRRT and non-CRRT strata, but these discrepancies were largely attenuated once individual concentrations were incorporated via Bayesian updating. These findings suggest that structural representation of renal replacement status, together with covariate availability and reconstruction rather than estimation paradigm alone, is a major determinant of predictive reliability when models are transported without re-estimation.

Our results are consistent with prior external evaluations demonstrating substantial inter-model variability in critically ill populations and dependence of predictive accuracy on renal support status and development cohort characteristics (25–27). Collectively, these data reinforce that model selection for MIPD should prioritize alignment between the development population, covariate structure, and the intended clinical application rather than relying solely on conventional prediction error metrics. (25)

### Limitations

This study has several limitations. First, the evaluation was conducted at a single center with a relatively small sample size and sparse sampling, particularly among patients receiving CRRT. Patients were sequentially enrolled through the parent ICU observational study, and inclusion in the present analysis was based on the availability of quantifiable meropenem concentrations, complete dosing histories, and clinical covariates required for external model implementation. Thus, the cohort reflects sample availability from a clinically relevant observational ICU study rather than random sampling or targeted selection of a specific subgroup. This limits the precision of subgroup-specific performance estimates and reduces the ability to generalize these findings across the full spectrum of critically ill patients. Therefore, these results should be interpreted as a focused external evaluation in a pneumonia-enriched ICU cohort rather than as a definitive ranking of meropenem population PK models across all critical care settings. Larger multicenter studies with richer sampling and broader representation of renal replacement modalities are warranted to validate CRRT-specific meropenem dosing models and confirm the transportability of candidate Bayesian priors (27).

Second, residual prediction error may reflect unmeasured or unmodeled clinical factors, including fluid balance, inflammatory status, or variability in extracorporeal support documentation, which were not explicitly incorporated into the evaluated models.

Third, variability summaries were derived from empirical Bayes estimates under each model. Because EBEs are susceptible to shrinkage in the setting of sparse data, especially when prior variability is large, comparisons of CV% across models and estimation paradigms should be interpreted qualitatively rather than as exact estimates of underlying between-subject variance.

Fourth, analyses were restricted to the implementation of published models without re-estimation of population parameters. This approach may underestimate the maximal achievable predictive performance of each model, but it was deliberately chosen to evaluate transportability when models are applied as fixed priors in external cohorts, consistent with contemporary external-evaluation guidance (6). In addition, translation of nonparametric models into parametric implementation preserves their originally reported variance structures, and reconstruction of certain covariates (e.g., MDRD- or CKD-EPI-based renal function estimates and residual diuresis) relied on available data and assumptions from the source publications; any misclassification or measurement error in these covariates could have impacted model performance in ways that are difficult to fully quantify.

### Conclusion

In this external evaluation, no CRRT-specified meropenem model achieved acceptable population-level performance in CRRT-treated patients, whereas incorporation of observed concentrations through Bayesian updating improved all CRRT-specified models to acceptable individual-level accuracy and precision. These findings indicate that population predictions alone are insufficient for clinically reliable meropenem forecasting in critically ill patients receiving CRRT and that Bayesian updating is essential in this setting. Model choice nevertheless remained important: the Rohani, Reed, and Shekar models demonstrated the most consistent predictive performance across the CRRT and non-CRRT subgroups. Together, these findings support meropenem MIPD strategies that pair Bayesian updating with physiologically relevant population priors aligned to renal support status and development population. Differences in clearance variability between native parametric priors and translated nonparametric-derived priors, together with the sensitivity of certain models to covariate reconstruction, further underscore that both structural and variability assumptions must be considered when transporting meropenem PK models into new critically ill populations.

## Data Availability

The clinical data underlying this study were collected per protocol (Northwestern University IRB STU0020408680 and STU00217256). Due to the sensitive nature of the patient data and institutional policies, data sets are not publicly available. De-identified data may be accessed by qualified researchers subject to a formal Data Use Agreement (DUA). Please contact a member of the SCRIPT study team (https://script.northwestern.edu/contact-us/) and the corresponding author to coordinate data access. To support transparency and reproducibility, the data analysis code has been made publicly available at: https://github.com/njrhodes/MEM_valid_public.

## SUPPLEMENTAL MATERIAL

Supplemental material aac.00458-26-s0001.pdf Tables S1 to S5; Fig. S1 to S4.

ASM does not own the copyrights to Supplemental Material that may be linked to, or accessed through, an article. The authors have granted ASM a non-exclusive, world-wide license to publish the Supplemental Material files. Please contact the corresponding author directly for reuse.

## References

[1] Sime FB, Roberts MS, Peake SL, Lipman J, Roberts JA. 2012. Does beta-lactam pharmacokinetic variability in critically ill patients justify therapeutic drug monitoring? a systematic review. Ann Intensive Care 2:35. doi:10.1186/2110-5820-2-35 22839761 PMC3460787

[2] Bergen PJ, Bulitta JB, Kirkpatrick CMJ, Rogers KE, McGregor MJ, Wallis SC, Paterson DL, Nation RL, Lipman J, Roberts JA, et al.. 2017. Substantial impact of altered pharmacokinetics in critically ill patients on the antibacterial effects of meropenem evaluated via the dynamic hollow-fiber infection model. Antimicrob Agents Chemother 61:e02642-16. doi:10.1128/AAC.02642-16 28264846 PMC5404522

[3] Roberts JA, Abdul-Aziz MH, Lipman J, Mouton JW, Vinks AA, Felton TW, Hope WW, Farkas A, Neely MN, Schentag JJ, International Society of Anti-Infective P, the P, Pharmacodynamics Study Group of the European Society of Clinical M, Infectious D. 2014. Individualised antibiotic dosing for patients who are critically ill: challenges and potential solutions. Lancet Infect Dis 14:498–509. doi:10.1016/S1473-3099(14)70036-2 24768475 PMC4181663

[4] Cheng Y, Wang CY, Li ZR, Pan Y, Liu MB, Jiao Z. 2021. Can population pharmacokinetics of antibiotics be extrapolated? implications of external evaluations. Clin Pharmacokinet 60:53–68. doi:10.1007/s40262-020-00937-4 32960439

[5] El Hassani M, Marsot A. 2023. External evaluation of population pharmacokinetic models for precision dosing: current state and knowledge gaps. Clin Pharmacokinet 62:533–540. doi:10.1007/s40262-023-01233-7 37004650

[6] El Hassani M, Marsot A. 2026. Guidance for external evaluation and selection of population pharmacokinetic models for precision dosing. Clin Pharmacokinet 65:1–9. doi:10.1007/s40262-025-01602-4 41343019

[7] Gijsen M, Dreesen E, Annaert P, Nicolai J, Debaveye Y, Wauters J, Spriet I. 2021. Meropenem pharmacokinetics and target attainment in critically ill patients are not affected by extracorporeal membrane oxygenation: a matched cohort analysis. Microorganisms 9:1310. doi:10.3390/microorganisms9061310 34208553 PMC8234236

[8] Idoate Grijalba AI, Aldaz Pastor A, Marquet P, Woillard JB. 2019. Evaluation of a non-parametric modelling for meropenem in critically ill patients using Monte Carlo simulation. Eur J Clin Pharmacol 75:1405–1414. doi:10.1007/s00228-019-02716-y 31338539

[9] Li C, Kuti JL, Nightingale CH, Nicolau DP. 2006. Population pharmacokinetic analysis and dosing regimen optimization of meropenem in adult patients. J Clin Pharmacol 46:1171–1178. doi:10.1177/0091270006291035 16988206

[10] O’Jeanson A, Larcher R, Le Souder C, Djebli N, Khier S. 2021. Population pharmacokinetics and pharmacodynamics of meropenem in critically ill patients: how to achieve best dosage regimen according to the clinical situation. Eur J Drug Metab Pharmacokinet 46:695–705. doi:10.1007/s13318-021-00709-w 34403127

[11] Reed G, Valadez A, Smith BJ, McCreary EK, Kline EG, Neely MN, Squires KM, Shields RK, Rhodes NJ. 2025. Population pharmacokinetics of meropenem-vaborbactam in acutely ill hospitalized patients with various degrees of renal dysfunction including continuous renal replacement therapy. Antimicrob Agents Chemother 69:e0010825. doi:10.1128/aac.00108-25 40455008 PMC12217465

[12] Rohani R, Yarnold PR, Scheetz MH, Neely MN, Kang M, Donnelly HK, Dedicatoria K, Nozick SH, Medernach RL, Hauser AR, et al.. 2023. Individual meropenem epithelial lining fluid and plasma PK/PD target attainment. Antimicrob Agents Chemother 67:e0072723. doi:10.1128/aac.00727-23 37975660 PMC10720524

[13] Shekar K, Fraser JF, Taccone FS, Welch S, Wallis SC, Mullany DV, Lipman J, Roberts JA, Investigators AES. 2014. The combined effects of extracorporeal membrane oxygenation and renal replacement therapy on meropenem pharmacokinetics: a matched cohort study. Crit Care 18:565. doi:10.1186/s13054-014-0565-2 25636084 PMC4302127

[14] SCRIPT Investigators. Successful Clinical Response in Pneumonia Therapy (SCRIPT). Northwestern University. Available from: https://script.northwestern.edu/database/. Retrieved 3030 JunJune 2026. Accessed , 3030 JunJune 2026

[15] Cockcroft DW, Gault MH. 1976. Prediction of creatinine clearance from serum creatinine. Nephron 16:31–41. doi:10.1159/000180580 1244564

[16] U.S. Department of Health and Human Services Food and Drug Administration Center for Drug Evaluation and Research (CDER) Center for Veterinary Medicine (CVM). 2018. Rockville, MD Food and Drug Administration (FDA)

[17] Avantor. Acetonitrile ≥99.9%, OmniSolv for LC-MS, Supelco. Available from: https://us.vwr.com/store/catalog/product.jsp?catalog_number=EM-AX0156-1. Retrieved 3030 JunJune 2026. Accessed , 3030 JunJune 2026

[18] Monolix 2024R1. 2024 Available from: https://www.simulations-plus.com/software/monolix/monolix

[19] Elassaiss-Schaap J, Heisterkamp S. 2009. Variability as constant coefficient of variation: can we right two decades in error? Petersburg, RU abstr PAGE

[20] Levey AS, Bosch JP, Lewis JB, Greene T, Rogers N, Roth D. 1999. A more accurate method to estimate glomerular filtration rate from serum creatinine: a new prediction equation. modification of diet in renal disease study group. Ann Intern Med 130:461–470. doi:10.7326/0003-4819-130-6-199903160-00002 10075613

[21] Levey AS, Stevens LA, Schmid CH, Zhang YL, Castro AF 3rd, Feldman HI, Kusek JW, Eggers P, Van Lente F, Greene T, Coresh J, Ckd EPI. 2009. A new equation to estimate glomerular filtration rate. Ann Intern Med 150:604–612. doi:10.7326/0003-4819-150-9-200905050-00006 19414839 PMC2763564

[22] Sheiner LB, Beal SL. 1981. Evaluation of methods for estimating population pharmacokinetic parameters. II. Biexponential model and experimental pharmacokinetic data. J Pharmacokinet Biopharm 9:635–651. doi:10.1007/BF01061030 7334463

[23] R Core Team. 2025. R: a language and environment for statistical computing. R Foundation for Statistical Computing. https://www.R-project.org.

[24] Keizer RJ, Ter Heine R, Frymoyer A, Lesko LJ, Mangat R, Goswami S. 2018. Model-informed precision dosing at the bedside: scientific challenges and opportunities. CPT Pharmacometrics Syst Pharmacol 7:785–787. doi:10.1002/psp4.12353 30255663 PMC6310898

[25] Bastida C, Escolà-Rodríguez A, Fernández S, Castro P, Soy D. 2025. External validation of population pharmacokinetic models for meropenem in critically ill adult patients. J Glob Antimicrob Resist 43:358–364. doi:10.1016/j.jgar.2025.05.015 40409494

[26] Schatz LM, Brinkmann A, Röhr A, Frey O, Greppmair S, Weinelt F, Zoller M, Scharf C, Hempel G, Liebchen U. 2023. Systematic evaluation of pharmacokinetic models for model-informed precision dosing of meropenem in critically Ill patients undergoing continuous renal replacement therapy. Antimicrob Agents Chemother 67:e0010423. doi:10.1128/aac.00104-23 37125925 PMC10190255

[27] Yang N, Wang J, Xie Y, Ding J, Wu C, Liu J, Pei Q. 2022. External evaluation of population pharmacokinetic models to inform precision dosing of meropenem in critically Ill patients. Front Pharmacol 13:838205. doi:10.3389/fphar.2022.838205 35662716 PMC9157771

