# Supplementary material for: Predictive accuracy of population meropenem models in critically ill pneumonia patients
Source: Antimicrob Agents Chemother. 2026 Jul 10;70(8):e00458-26. doi: 10.1128/aac.00458-26 (PMC13436005; doi:10.1128/aac.00458-26)
Supplement: Supplementary file 1 [file aac.00458-26-s0001.pdf]

Supplementary Materials

Tables

Table S1. Fixed-effect parameters, between-subject variability, and residual unexplained variability for the meropenem population PK models

Parameter	*Theta (θ)*	*Omega (ω)*	*Residual unexplained variability (RUV)*
**Gijsen**			
CL (L/h)	14.7	0.44	
V1 (central volume, L)	25.6	0.5672	
Q (intercompartmental CL, L/h)	5.51	---	
V2 (peripheral volume, L)	8.02	---	
CKD-EPI exponent on CL	1.29	---	
WT exponent on CL	0.75 (FIX)	---	
WT exponent on V1	1.00 (FIX)	---	
WT exponent on Q	0.75 (FIX)	---	
WT exponent on V2	1.00 (FIX)	---	
Residual error model			Proportional; 0.288
**Grijalba**			
Ke1 (baseline Ke at median CL_{CR}, h^{-1})	0.345	0.55	
Ke2 (CL_{CR} power term on Ke)	0.725	0.71	
V (central volume, L)	25.1	0.64	
F_free (prop; CT -> CF scaling)	0.824	0.32	
Residual error model			Proportional; 0.25<br>Additive: 0.05
**Li**			
CL (L/h)	14.6	0.343	
V1 (central volume, L)	10.8	0.378	
Q (intercompartmental CL, L/h)	18.6	0.539	
V2 (peripheral volume, L)	12.6	0.319	
CRCL exponent on CL	0.62	---	
AGE exponent on CL	-0.34	---	
WT exponent on V1	0.99	---	

Residual error model			Combined additive + proportional; 0.469, 0.188
O'Jeanson			
CL (L/h)	1.36	0.301	
GFR effect on CL (slope)	0.058	---	
DIA_C (dialysis clearance term)	6.38	---	
RFS (renal function slope)	13.9	---	
RD (renal dysfunction factor)	0.6	---	
V (central volume, L)	43.9	0.755	
Residual error model			Proportional; 0.321
Reed			
CL (non CRRT, L/h)	10.585	0.375	
CL (residual, L/h)	2.095	0.418	
K12 (h^{-1})	0.767	0.584	
K20 (filter elimination, h^{-1})	4.442	0.506	
K23 (h^{-1})	29.269	0.295	
K31 (h^{-1})	1.671	0.918	
V (L, V/80 kg)	27.391	0.371	
Residual error model			Combined additive + proportional; 0.57, 0.0855
Rohani			
V (central volume, L)	29.7	0.67	
CL1 (non-RRT clearance, L/h)	8.5	0.48	
CL2 (iHD clearance, L/h)	9.5	0.67	
CL3 (CRRT clearance per unit FLOW, L/h)	2.2	0.83	
pwr (CRCL exponent on CL1)	0.75	0.70	
Residual error model			Combined additive + proportional; 0.496, 0.0744
Shekar			
V1 (central volume, L)	18.7	0.436	
CL1 (L/h)	5.1	0.486	

Q1 (intercompartmental clearance, L/h)	21	---	
V2 (peripheral volume, L)	13.2	0.281	
pwr (CrCL exponent on CL)	1.89	---	
Residual error model			Combined additive + proportional; 2.3, 0.137

Table legend: Fixed-effect parameters are reported as theta (**θ**) and between-subject variability as omega (**ω**). Residual unexplained variability (RUV) terms reflect the residual error models implemented in Monolix for external validation. When residual error was reported in the source publication as CV%, the corresponding Monolix proportional error term is shown.

Table S2. Predictive Performance of population PK models in CRRT and Non-CRRT Cohort

	Models	Population		Individual	
		rMPE (%)	rMdAPE (%)	rMPE (%)	rMdAPE (%)
CRRT Group Observations (n = 31)					
Parametric Models	O'Jeanson (fixed RD)	-39.7	45.1	-8.9	14.2
	O'Jeanson (individual RD)	-35.2	41.1	-8.2	13.5
	Shekar	-25.7	34.0	3.4	11.8
Nonparametric Models	Reed	-26.6	33.7	1.7	5.0
	Rohani	-28.6	37.1	3.6	5.1
Non-CRRT Group Observations (n = 43)					
Parametric Models	Gijsen	-3.5	42.0	1.3	19.2
	Li	-53.6	51.1	-3.4	12.8
	O'Jeanson (fixed RD)	-4.1	18.6	-6.6	13.6
	O'Jeanson (individual RD)	-21.5	28.3	-8.0	14.8
	Shekar	-8.5	20.4	0.5	11.0
Nonparametric Models	Grijalba	-24.8	31.1	2.0	7.4
	Reed	18.7	28.6	4.5	9.0
	Rohani	-7.7	18.0	1.7	7.9

Table S3. Characteristics of Published Meropenem Pharmacokinetic Models Evaluated

Model (Reference)	***Estimation paradigm***	***Development population***	***Structural model***	***Renal function scaling***	***RRT Handling***	***Eligible analyses***
Rohani et al. (2023)	Nonparametric (Pmetrics)	Critically ill ICU pneumonia patients (plasma ± ELF)	1-compartment (plasma)	Nonlinear CrCL scaling with power function	Flow-dependent CRRT clearance term	Pooled, CRRT, non-CRRT
Reed et al. (2025)	Nonparametric (Pmetrics)	Acutely ill hospitalized adult patients with renal dysfunction (including CRRT)	Hybrid: 1-compartment (non-CRRT); 3-compartment CRRT model	BSA-adjusted CrCL scaling in non-CRRT; CRRT-specified clearance term	Binary CRRT indicator with fixed CRRT clearance	Pooled, CRRT, non-CRRT
Grijalba et al. (2019)	Nonparametric (Pmetrics)	Critically ill ICU patients	1-compartment (free concentration output)	CKD-EPI-based elimination via power function centered at cohort median	None	Non-CRRT only
Li et al. (2006)	Parametric (NONMEM)	Adult hospitalized patients with bacterial infections	2-compartment IV model	CrCL- and age-dependent clearance; weight-scaled central volume	None	Non-CRRT only
Gijsen et al. (2021)	Parametric (NONMEM)	Critically ill ICU patients with and without ECMO	2-compartment IV model	CKD-EPI-based clearance with allometric weight scaling	None	Non-CRRT only
O'Jeanson et al. (2021)	Parametric (NONMEM)	Critically ill ICU patients with heterogeneous renal function	1-compartment IV model	State-based renal function model incorporating	Categorical renal support states	Pooled, CRRT, non-CRRT

				MDRD and residual diuresis	(CRRT/renal failure)	
Shekar et al. (2014)	Parametric (NONMEM)	Critically ill adults receiving ECMO ± RRT	2-compartment IV model	CrCL-dependent clearance in non-CRRT; distinct CRRT-associated clearance component	Binary CRRT-associated clearance term	Pooled, CRRT, non-CRRT

Table legend: Summary of published meropenem population PK models included for external evaluation. Structural and renal handling descriptions reflect the original publications. “RRT handling” indicates whether renal replacement therapy (RRT), including continuous renal replacement therapy (CRRT), was structurally incorporated into the model. “Eligible analyses” denotes the evaluation subsets in which each model was applied in the present study, based on structural compatibility with CRRT.

Table S4. Renal Clearance Equations Used for Model Translation

	Models	*Population Renal Clearance Equation*
Parametric Models	Gijsen	$CL = \theta_{CL} \times \left(\frac{WT}{70}\right)^{0.75} \times \left(\frac{CKD-EPI}{105}\right)^{1.29}$
	Li	$CL = \theta_{CL} \times \left(\frac{CL_{CR}}{83}\right)^{0.62} \times \left(\frac{AGE}{35}\right)^{-0.34}$
	O'Jeanson (fixed RD)	$CL = I_{CRRT} \times \theta_{DIA} + I_{RFS} \times \theta_{RFS} \times I_{STD} \times (\theta_{CL} + \theta_{GFR} \times MDRD)$
	O'Jeanson (individual RD)	$CL = I_{CRRT} \times \theta_{DIA\_C} + I_{RFS} \times \theta_{RFS} \times I_{STD}(\theta_{CL} + \theta_{GFR} \times MDRD) + \theta_{RD} \times \left(\frac{DIUR}{845} - 1\right)$
	Shekar	$CL = \theta_{CL} \times CRRT + \theta_{CL} \left(\frac{CRCL}{100}\right)^{pwr} \times (1 - CRRT)$
Nonparametric Models	Grijalba	$CL = \theta_{CL} \times \left(\frac{CKD-EPI}{82.9}\right)^{pwr}$
	Reed	$CL = CL_{nonCRRT} \times \left(\frac{CrCL_{BSA}}{120}\right) \times (1 - CRRT) + CL_{CRRT} \times (CRRT)$
	Rohani	$CL = CL_1 \times \left(\frac{CrCL}{120}\right)^{pwr} \times (1 - CRRT) \times (1 - HD) + CL_2 \times HD + CL_3 \times CRRT \times \left(\frac{FLOW}{1000}\right)$

Table legend: Renal clearance equations as implemented in Monolix for external validation. Structural forms reflect original publications; notation was harmonized for clarity without altering functional relationships. θ_CL denotes typical clearance. CrCL represents creatinine clearance (mL/min). CRRT is a binary indicator (0 = off, 1 = on). FLOW represents CRRT effluent rate (mL/h). Normalization constants reflect values reported in the original publications or corresponding cohort medians when required for implementation.

Table S5. Comparison of External Evaluation Cohort With Development Populations of Evaluated Meropenem Pharmacokinetic Models

Model source	*N/PK samples*	*Age*	*Sex*	*Body size*	*Renal function*	*RRT/CRRT/ECMO exposure*	*Illness severity*
Current external evaluation cohort	17 patients; 74 plasma concentrations	64.4 ± 15.9	Male 12/17 (71%); Female 5/17 (29%)	Weight 80.6 ± 22.2 kg, BSA 1.9 ± 0.3 m^2	SCr 1.7 ± 1.2 mg/dL; CrCl 76.8 ± 66.2 mL/min; CKD-EPI 59.2 ± 34.5 mL/min/1.73 m^2; MDRD 67.5 ± 13.2 mL/min/1.73 m^2; urine output 1240.1 ± 1593.1 mL/day	CRRT 6/17 (35%)	SOFA 11.7 ± 4.2
Rohani et al.	67 patients in PK cohort	57 ± 14.6 years	Male 43/67 (64%)	Weight 79.5 ± 17.4 kg; height 171 ± 9.1 cm; BSA 1.9 ± 0.2 m^2	SCr 1.1 ± 0.96 mg/dL; CrCl 128 ± 82 mL/min; indexed CrCl 115 ± 72 mL/min/1.73 m^2	ECMO 7/67 (10%); any CRRT 22/67 (33%); CVVH 4/67 (6%); CVVHDF 20/67 (30%); iHD 7/67 (10%)	Not reported
Reed et al.	18 patients; 83 plasma samples	54.7 ± 14.4 years	Male 8/18 (44%); Female 10/18 (56%)	TBW 90.7 ± 32.6 kg; BSA 2.02 ± 0.36 m^2	SCr 0.99 ± 0.74 mg/dL; baseline CrCl 123.3 ± 103.7 mL/min/1.73 m^2	AKI not on CRRT 2/18 (11.1%); CRRT 3/18 (16.7%)	Not reported
Grijalba et al.	80 patients; 154 total concentrations and 29 free concentrations	63.26 ± 15.07 years	Male 49/80 (61.25%)	Height 168 cm (144 – 192); real weight 72.76 ±19.17 kg; BMI 25.74 ± 5.93 kg/m^2; BSA 1.78 ± 0.28 m^2	Creatinine at meropenem start 0.9 mg/dL (0.2 – 5.50); clearance at meropenem start 82.91 mL/min/1.73 m^2 (14.33 – 125.66)	RRT excluded	SOFA on admission 4 (0 – 14); SAPS III 66.5 (39 – 96); worst SOFA 7 (1 – 14)
Li et al.	79 patients; 341 concentrations; 1 – 12 concentrations per patient	39.6 ± 18.2 years; median 35 (18 – 93)	Male/Female 61/18	Weight 73.0 ± 16.1 kg, median 70 (40.6 – 127); height 169.5 ± 8.7 cm; median 170 (147 – 185.4)	Serum creatinine 1.1 ± 0.8 mg/dL; median 1.0 (0.4 – 6.9)	Not reported; no specific CRRT-specific clearance structure	Not reported
Gijsen et al.	25 patients; 27 dosing intervals, popPK based on 24 non-CRRT matched dosing intervals from 22 patients	ECMO: 47 years [39 – 46]; non-ECMO: 68 years [63 – 71]	ECMO: Male 9/14 (64%); non-ECMO: Male 7/11 (64%)	ECMO: weight 84 kg [54.6 – 90]; non-ECMO: 70 kg [61 – 77.5]	eGFR CKD-EPI on PK Day: ECMO 106 [80 – 117], non-ECMO 97 [92 – 106] mL/min/1.73	ECMO 14 patients; VA-ECMO 2/14 (14%); CRRT 3/15 ECMO dosing intervals; no CRRT in non-ECMO cohort; CRRT intervals excluded from popPK model	SOFA admission: ECMO 9 [6 – 12], non-ECMO 8 [6 – 11]; APACHE II: ECMO 21 [18 – 23], non-ECMO 21 [16 – 27]; SOFA

					m^2; mCrCl24h: ECMO 57 [47 – 120], non-ECMO 70 [46 – 112]		on PK Day: ECMO 11 [8 – 14], non-ECMO 8 [5 – 9]
O'Jeanson et al.	58 patients; 115 serum concentrations included after BLQ/outlier exclusions	62 ±14.7 years; median 64 (18 – 85)	Male 41/58 (71%); Female 17/58 (29%)	Weight 72 ± 17.2 kg; median 68 (39.5 – 124); BMI 25 ± 6.1 kg/m^2	SCr 154 ± 109 µmol/L; CrCl CG 72 ± 60 mL/min, median 52 (10 – 376); MDRD GFR 78 ± 84 mL/min/1.73 m^2, median 49 (7 – 567); residual diuresis 1316 ± 1422 mL/day, median 845 (0 – 7300)	RRT 26/58; continuous RRT 6; semi-continuous RRT 10; combination RRT techniques/plasmapheresis 10	Not reported
Shekar et al.	21 total; controls no RRT n = 5; controls RRT = 5; ECMO no RRT n = 6; ECMO RRT n = 5	Controls no RRT: 55 (48 – 61); controls RRT: 56 (46 – 66); ECMO no RRT: 29 (16 – 46); ECMO RRT: 38 (23 – 56) years	Male/Female: 3/2; 3/2; 1/5; 3/2 by subgroup	Weight: 80 (75 – 85); 70 (60 – 100); 69 (60 – 80); 70 (70 – 76) kg by subgroup	CrCl available in non-RRT groups only: controls no RRT: 106 (98 – 127) mL/min; ECMO no RRT 108 (65 – 183) mL/min; RRT groups reported as not applicable because patients were RRT dependent	ECMO no RRT n = 6; ECMO RRT n = 5; VA/VV ECMO 3/3 and 2/3 in ECMO no RRT and RRT groups, respectively; RRT modes: CVVH in controls and EDD-f in ECMO	Day 1 SOFA: controls no RRT 3 (3 – 4); controls RRT 15 (14 – 16); ECMO no RRT 9 (7 – 14); ECMO RRT 16 (13 – 17)

Table legend: Clinical and model-relevant descriptors of the current external evaluation cohort compared with the development populations of the evaluated meropenem pharmacokinetic models. Values are presented as reported in the source publication and may therefore differ in summary format across studies. "Not reported" indicates that the descriptor was not available in the original publication **Abbreviations:** AKI, acute kidney injury; APACHE, Acute Physiology and Chornic Halth Evaluation; BLQ, below the limit of quantification; BSA, body surface area; CKD-EPI, Chronic Kidney Disease Epidemiology Collaboration; CrCl, creatinine clearance; CRRT, continuous renal replacement therapy; CVVH, continuous venovenous hemofiltration; CVVHDF, continuous venovenous hemodiafiltration; ECMO, extracorporeal membrane oxygenation; iHD, intermittent hemodialysis; MDRD, Modification of Diet in Renal Disease; PK, pharmacokinetic; RRT,

renal replacement therapy; SAPS, Simplified Acute Physiology Score; SCr, serum creatinine; SOFA, Sequential Organ Failure Assessment; TBW, total body weight.

Figure S1. Observed versus predicted meropenem concentrations in patients receiving continuous renal replacement therapy (CRRT)

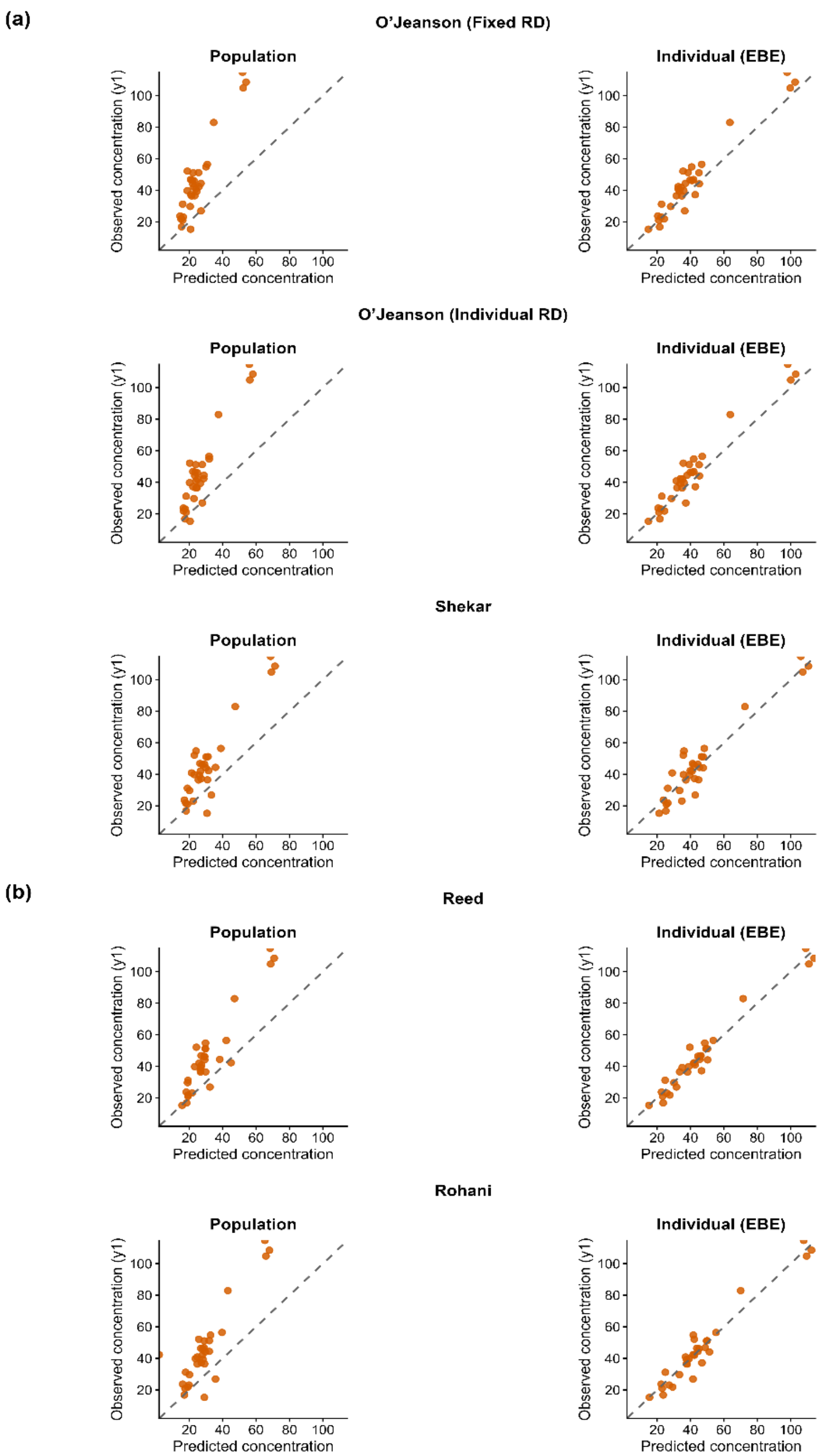

Figure legend: Population predictions (left) and individual Bayesian predictions (right) are shown for published population PK models incorporating CRRT-specified clearance, with parametric models displayed in panel (a) and nonparametric models in panel (b). The dashed line represents the line of identity. Points are colored by patient identifier.

Figure S2. Observed versus predicted meropenem concentrations in non-renal replacement (non-CRRT) patients

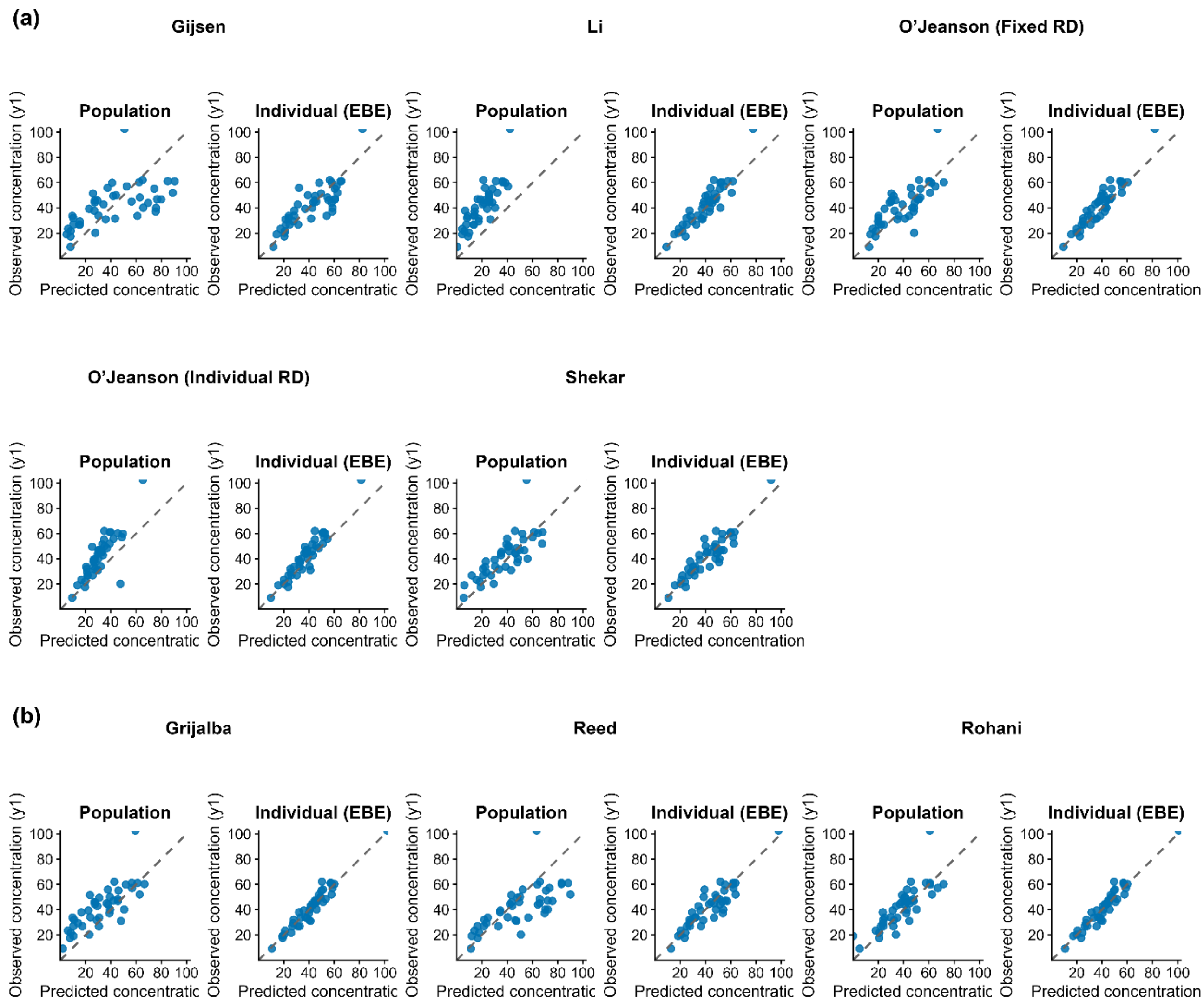

Figure legend: For each model, population predictions (left) and individual Bayesian predictions (right) are shown, with parametric models grouped in panel (a) and nonparametric models grouped in panel (b). The dashed line represents the line of identity. Points are colored by patient identifier.

Figure S3. Individual Weighted Residuals (IWRES) Versus Individual Predictions Across Evaluated Meropenem Population PK Models

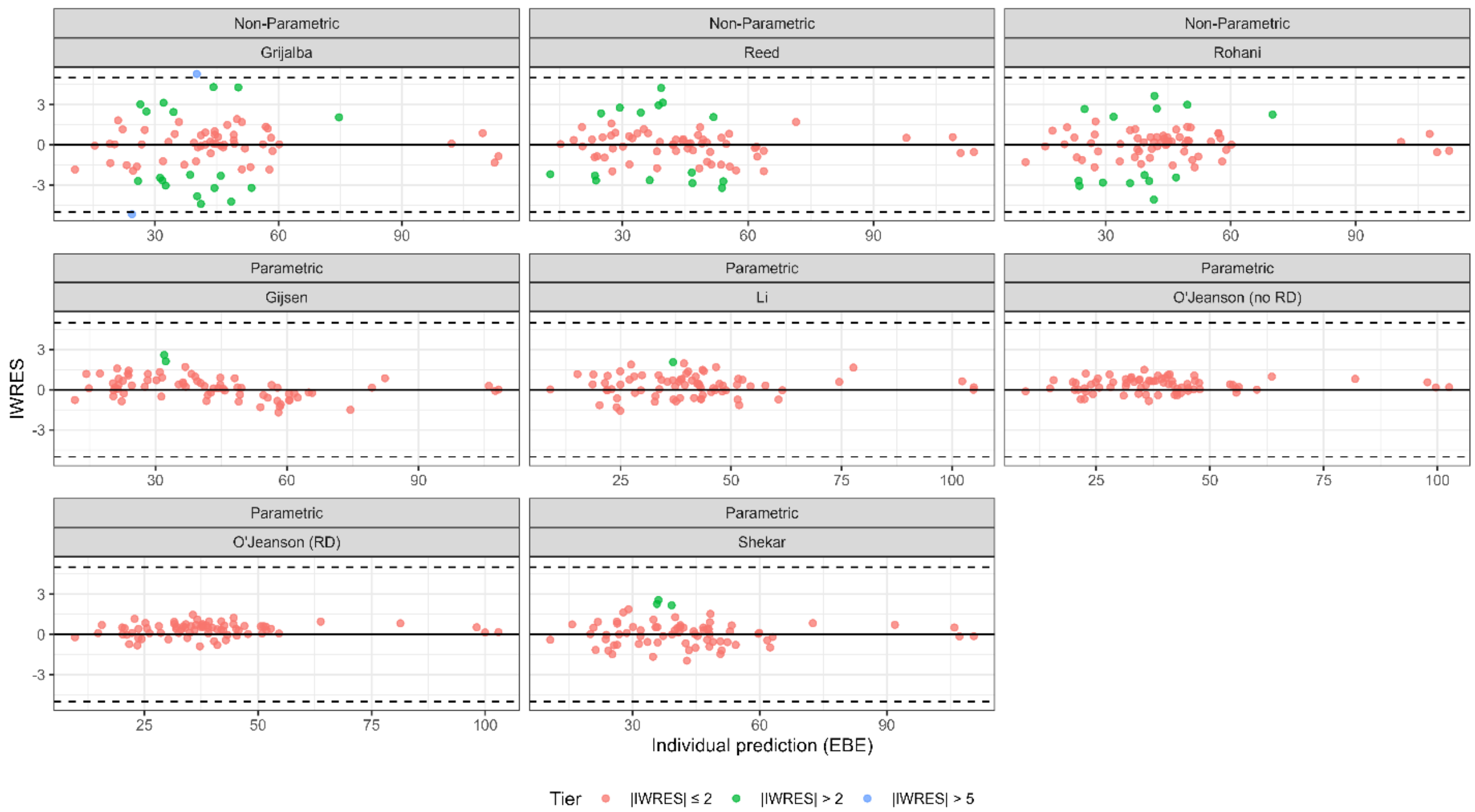

Figure legend: The solid line represents zero residual error; dashed lines denote ± 5 IWRES. Points are colored by residual magnitude.

Figure S4. Individual Weighted Residuals (IWRES) Versus Time Across Evaluated Models

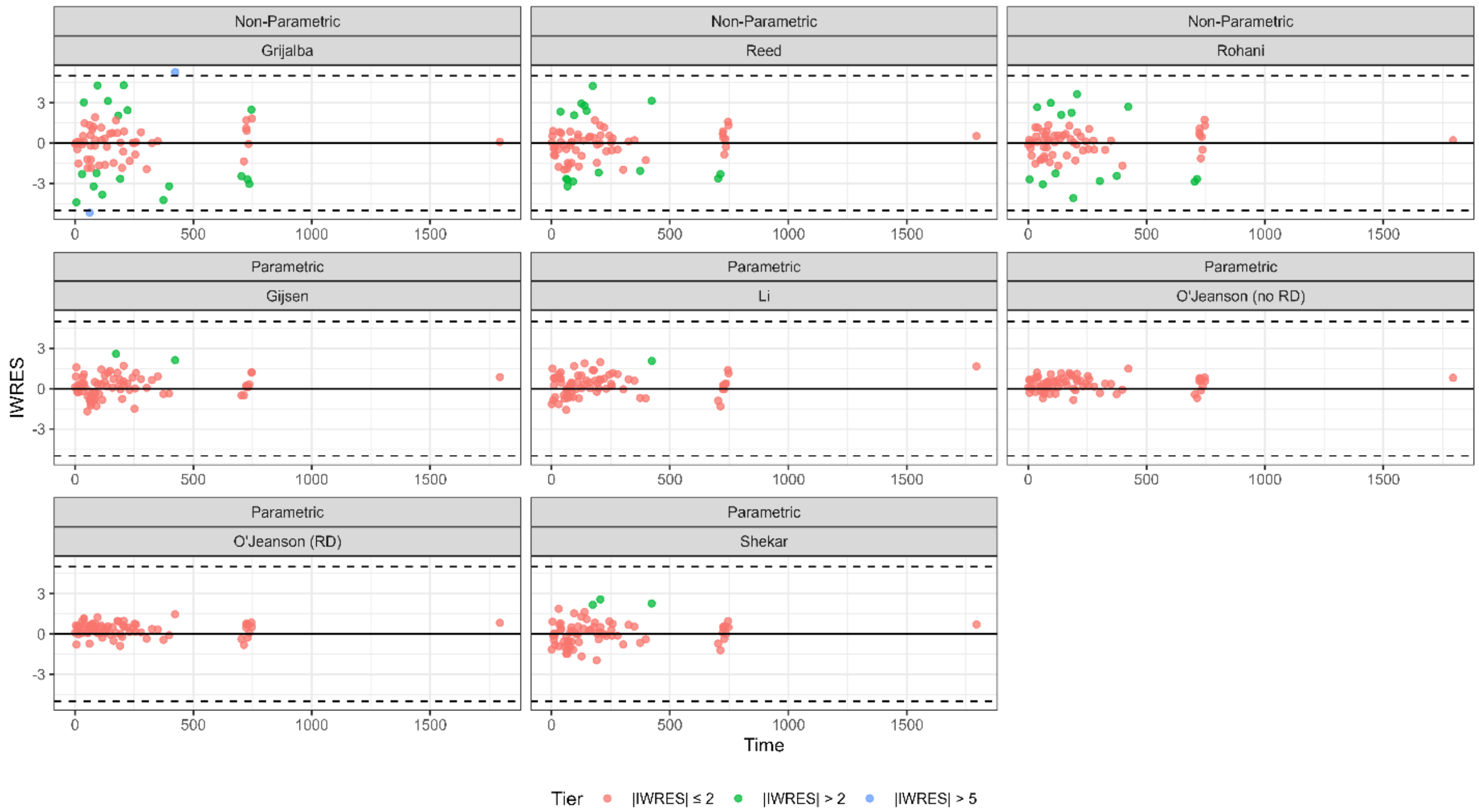

Figure legend: The solid line represents zero residual error; dashed lines denote ± 5 IWRES. Points are colored by residual magnitude.
